# Education mediates the relationship of parental socioeconomic status with subjective adult oral health

**DOI:** 10.1186/s12955-023-02169-z

**Published:** 2023-08-10

**Authors:** Faten Haider, Eduardo Bernabé, Elsa Karina Delgado-Angulo

**Affiliations:** 1 Faculty of Dentistry, Oral & Craniofacial Sciences, King’s College London, London, UK; 2 Centre for Dental Public Health and Primary Care, Institute of Dentistry, Queen Mary University of London, Turner street, E1 2AD London, UK; 3 Facultad de Estomatología, Universidad Peruana Cayetano Heredia, Lima, Perú

**Keywords:** Oral health, Education, Life course perspective, Socioeconomic factors, Mediation

## Abstract

**Background:**

Evidence shows that both socioeconomic status (SES) during childhood and education are associated with adult oral health. However, whether the range of opportunities families have regarding their children’s education mediate the effect of childhood disadvantage on oral health later in life remains unknown. The aim of this study was to evaluate the mediating role of education in the association between parental SES and subjective oral health status in middle adulthood.

**Methods:**

Data from 6703 members of the British Cohort Study 1970 were analyzed. Parental SES was measured using the 7-class National Statistics Socio-Economic Classification (NS-SEC) at age 10 years. Five measures of education (type of high school, highest qualification, age left full-time education, status of institution and field of study) were obtained from ages 16 and 42 years. Subjective oral health was measured with a single global item at age 46 years. Causal mediation analysis was performed, using a weighting-based approach, to evaluate how much of the effect of parental SES on subjective oral health was mediated by the measures of education separately and jointly.

**Results:**

Overall, 23.6% of individuals reported poor oral health. Parental SES was associated with every measure of education, and they were also associated with subjective oral health in regression models adjusted for confounders. The effect of parental SES on subjective oral health was partially mediated by each measure of education, with a proportion mediated of 53.2% for the institution status, 46.5% for the field of study, 42.8% for the school type, 38.9% for the highest qualification earned and 38.4% for the age when full-time education was discontinued. The proportion of the effect of parental SES on subjective oral health jointly mediated by all measures of education was 81.1%.

**Conclusion:**

This study found a substantial mediating role of education in the association between parental SES and subjective oral health in middle adulthood.

## Introduction

Several birth cohort studies have shown that low parental socioeconomic status (SES) is a strong predictor of poor clinical and subjective oral health in adulthood [1–5], with materialist, psychosocial, behavioural and lifecourse explanations commonly proposed as underlying pathways [6]. Within the life course perspective [7], a key question to address is whether low parental SES influences adult health independent of educational attainment or whether educational attainment has a mediating role in the association between parental SES and adult oral health (the so-called indirect and direct effects of parental SES on adult oral health). On one hand, educational attainment is associated with both future health and economic outcomes [8–11]. Higher education is associated with stronger neural development and slower biological ageing; a stronger sense of control over life that enables coping with life’s challenges; better knowledge, choices and access to healthier lifestyles and healthcare; and greater socioeconomic resources [12–14]. On the other hand, educational attainment is strongly associated with socioeconomic circumstances during childhood [15]. Low parental SES can limit academic success by reducing childcare options, parent–child interactions, access to books and computers at home, access to quality schooling, and participation in the school community [8, 16]. Parental SES is associated with differences in individuals’ academic performance (primary effects) as well as differences in the educational opportunities that individuals have, given performance (secondary effects) [17].

There is vast evidence that education is associated with adult oral health [18]. However, the studies in this review did not account for parental SES, which is a well-established determinant of both education and oral health. What is more, most studies focused on the highest qualification earned by individuals despite it is becoming increasingly evident that other facets of education, such as the status of the institution attended and the field of study, may be relevant to fully understand the role of education in health status [19]. A recent study showed that attending private schools and higher-status universities were associated with multiple favourable health behaviours, lower body mass index and better subjective health in midlife [20]. Another study showed that the timing of educational credentials was associated with physical health; that is, earning a first degree at younger ages was a stronger predictor of health than earning the same degree at later ages [21]. What is missing in the oral epidemiology literature is a comprehensive assessment of whether the range of opportunities families have regarding their children’s education can influence their oral health later in life. Understanding the mediating role of education in the relationship between parental SES and oral health can inform targets for social and economic policies that support high quality public education and reduce health inequalities. The aim of this study was to evaluate the mediating role of education in the association between parental SES and subjective oral health in middle adulthood.

## Methods

### Study population

This study used data from the 1970 British Cohort Study (BCS70), which follows the lives of 17,196 individuals born in England, Wales, and Scotland during the first week of April in 1970. Cohort members have been invited to participate in 11 waves of data collection to date, with the most recent wave in 2021 when cohort members were 51 years old [22]. Data for this study were taken from multiple waves, namely parental SES and cohort member’s demographic factors (wave 3, age 10), measures of education (from wave 4, age 16, to wave 9, age 42) and oral health (wave 10, age 46). Seventy percent of the original cohort was retained by age 46 years. There is evidence that retained cohort members are wealthier and healthier than participants lost to follow-up [23].

A total of 8581 individuals participated in BCS70 wave 10. Of them, 1878 were excluded for having missing data on parental SES (*n* = 1322), measures of education (*n* = 685), ethnicity (*n* = 64) and oral health (*n* = 5). The final sample for this study included 6703 cohort members.

### Measures

**Fig. 1:**
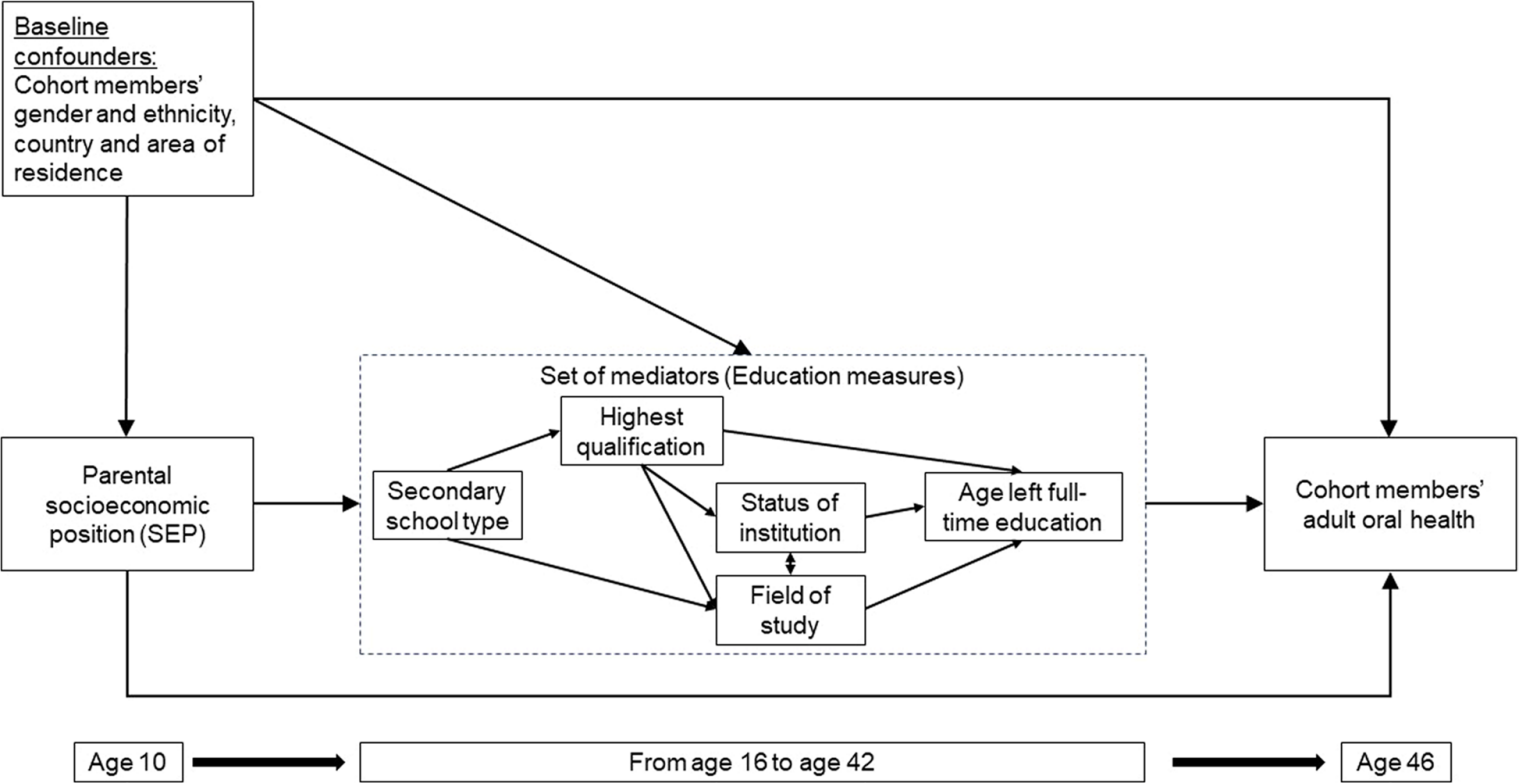
Directed acyclic graph for the hypothesised relationships between parental SES (exposure), measures of education (mediators) and subjective oral health (outcome), including all potential common causes (confounders) at baseline

The hypothesised relationships between parental SES, education and subjective oral health, including all potential common causes (confounders) at baseline, are shown in a directed acyclic graph (DAG, Fig. 1). The outcome was poor subjective oral health, which was collected with the question ‘would you say that your dental health (mouth, teeth, and/or dentures) is … excellent, very good, good, fair or poor?’ Those who answered fair or poor were considered as having poor subjective oral health.

The exposure was parental SES at age 10 years because the outdated Registrar General’s Social Class was rebased to the official National Statistics Socioeconomic Classification (NS-SEC) for that specific BCS70 wave only [24]. The full seven NS-SEC groups were used, namely (i) higher managerial, administrative and professional occupations (highest class, reference category), (ii) lower managerial, administrative and professional occupations, (iii) intermediate occupations, (iv) small employers and own account workers, (v) lower supervisory and technical occupations, (vi) semi-routine occupations and (vii) routine occupations (lowest class).

Education was the mediator evaluated with five measures. The type of high school attended (from age 11 to 16) was derived from interviews with school headmasters and from census records at 16 years or recalled at 42 years if not available [20, 25]. School type was categorised as private (academically selective schools for those able to pay), grammar (state academically selective schools), secondary modern (state schools for those not selected for grammars), comprehensive (all-abilities state schools, reference category) and special education needs (state schools for children with learning problems and disabilities). The age at which full-time education was discontinued (and cohort members did not return to education afterwards), the highest academic qualification, and the field of study and status of the institution for those with a university degree were recalled at age 42. The National Vocational Qualification (NVQ) system was used to classify individuals according to their highest qualification as: no degree, level 1 (equivalent to 3–4 GCSE grades 1–3), level 2 (4–5 GCSE grades 4–9), level 3 (2 A Levels), level 4 (higher education certificate/technical qualifications), and level 5 (higher education diploma/foundation degree). For degree level qualifications, the field of study was classified as Science, Technology, Engineering, Maths (STEM), Law, Economics, Management (LEM) and Other Social Sciences, Arts and Humanities (OSSAH) [26, 27], and the status of the institution attended was classified as either higher status (Russell group) or normal status (all other institutions). The Russell group is a self-selected group representing 24 purportedly leading universities [20, 28].

The following baseline demographic characteristics of cohort members were also included in the analysis as confounders of the association between parental SES and adult oral health: sex, ethnicity (white, non-white), country (England, Wales, Scotland) and area of residence (urban, rural).

### Data analysis

Data management and regression modelling were performed in Stata. First, parental SES groups were compared in terms of their demographic characteristics using the Chi-squared test. Thereafter, two set of regression models were fitted. The first set of models tested the association between the exposure (parental SES) and each mediator (education measures) adjusted for confounders (sex, ethnicity, country and area of residence). Categorical measures of education (type of high school, highest qualification, status of institution and field of study) were modelled using multinomial regression whereas the numerical measure of education (age left full-time education) was modelled using linear regression. The second set of models tested the association between each mediator (education measures) and the outcome (subjective oral health) adjusted for parental SES and confounders. Subjective oral health was modelled using binary logistic regression.

Causal mediation analysis, which builds on a counterfactual framework, was used to evaluate how much of the effect of parental SES on subjective oral health was mediated by the measures of education [29]. In the odds ratio scale [30], the total effect (TE) of parental SES is the relative difference in the odds of poor subjective oral health between those in the lowest and highest SES groups, adjusted for confounders. The TE decomposes into the natural indirect effect (NIE, which goes through the measures of education) and the natural direct effect (NDE, which goes through pathways not involving any measures of education). The NDE expresses how much the odds of poor subjective oral health would change if parental SES were set to the lowest group, versus the highest, but the measure of education was set to the level that would have naturally occurred in the highest SES group. The NIE expresses how much the odds of poor subjective oral health would change if parental SES were fixed at the highest group, but the measure of education was changed from what it would have been in the lowest versus the highest SES group. The proportion mediated was estimated to quantify the extent to which the TE of parental SES on subjective oral health operates through the measure(s) of education [30]. These natural effects are valid estimates provided that (i) the set of confounders suffice to control for exposure-outcome, mediator-outcome and exposure-mediator confounding, and (ii) the regression models are correctly specified [31]. We used a weighting-based approach, which required no models for the mediators and allowed estimating their individual and joint mediating effects, to overcome the issue of model identifiability due to the presence of exposure-induced mediator-outcome confounding in our DAG [29, 31]. We first regressed parental SES on confounders using ordinal logistic regression. Next, for each measure of education separately (and then all measures jointly), we regressed subjective oral health on parental SES, the measure(s) of education, an interaction term between parental SES and the measure(s) of education, and confounders using binary logistic regression. The interaction term was included to allow for the decomposition of the TE into NDE and NIE in the presence of exposure-mediator interaction [32]. All estimates with bootstrapped confidence intervals were derived using the CMAverse package in R [33].

Sensitivity analysis was carried out to evaluate the potential for bias from unmeasured confounding [34]. The E-value was used to estimate the minimum strength of association that an unmeasured confounder would need to have with exposure and outcome, and with mediator and outcome, conditional on the measured confounders, to fully explain away the NDE and NIE, respectively [35].

## Results

Data from 6703 individuals were available for analysis. Differences between individuals included in the analysis and those excluded due to missing values on relevant variables were found. Female, White, Scottish and urban individuals, those with higher parental SES and education and better oral health were more likely to be in the study sample. Table 1 compares the characteristics of the parental SES groups. There were more individuals from ethnic minorities and rural areas in the lower SES groups. Almost a quarter of cohort members (23.6%) reported poor subjective oral health.

**Table 1:** Comparison of the characteristics of groups defined according to parental SES

	**Higher managerial**		**Lower managerial**		**Intermediate occupations**		**Small employers**		**Lower supervisory**		**Semi-routine occupations**		**Routine occupations**		***P* value**^a^
	**n**	**%**	**n**	**%**	**n**	**%**	**n**	**%**	**n**	**%**	**n**	**%**	**n**	**%**	
*Sex*															0.107
Men	444	48.7	619	46.1	565	47.8	366	52.0	372	46.6	456	44.9	358	47.9	
Women	468	51.3	724	53.9	617	52.2	338	48.0	427	53.4	560	55.1	389	52.1	
*Ethnicity*															< 0.001
White	888	97.4	1,298	96.7	1144	96.8	665	94.5	766	95.9	953	93.8	699	93.6	
Non-white	24	2.6	45	3.4	38	3.2	39	5.5	33	4.1	63	6.2	48	6.4	
*Country of residence*															0.089
England	794	87.1	1,159	86.3	1032	87.3	581	82.5	700	87.6	873	85.9	624	83.5	
Scotland	73	8.0	103	7.7	96	8.1	68	9.7	57	7.1	88	8.7	72	9.6	
Wales	45	4.9	81	6.0	54	4.6	55	7.8	42	5.3	55	5.4	51	6.8	
*Residence area*															< 0.001
Urban	611	67.0	903	67.2	836	70.7	445	63.2	584	73.1	739	72.7	548	73.4	
Rural	301	33.0	440	32.8	346	29.3	259	36.8	215	26.9	277	27.3	199	26.6	

*NS-SEC* National Statistics Socio-Economic Classification

Chi-squared test was used for comparison between parental SES groups

Parental SES was positively associated with every measure of education (Table 2). Individuals raised in lower parental SES left full-time education at younger ages and had lower odds of attending grammar and private schools, obtaining NVQ3, NVQ4 and NVQ5 qualifications, attending normal and higher status institutions and studying STEM, LEM and OSSAH courses. Furthermore, every measure of education was associated with subjective oral health. The odds of poor subjective oral health were lower among individuals who attended grammar and private schools than those who attended comprehensive school, among those with higher qualifications attained, among those who studied in normal and higher status institution and those who studied STEM, LEM and OSSAH courses than those with no degree, and among those who finished full-time education at a younger age (Table 3).

**Table 2:** Regression models for the association of parental SES (NS-SEC) with five different measures of education (*n* = 6703)

	Type of high school				Age left full-time education
	Grammar versus Comprehensive	Secondary Modern vs Comprehensive	Private vs Comprehensive	Special needs vs Comprehensive	
	OR [95% CI]	OR [95% CI]	OR [95% CI]	OR [95% CI]	Coef. [95% CI]
Higher managerial	1.00 [Reference]	1.00 [Reference]	1.00 [Reference]	1.00 [Reference]	0.00 [Reference]
Lower managerial	0.62 [0.44, 0.88]*	1.01 [0.69, 1.47]	0.46 [0.35, 0.59]*	2.48 [0.52, 11.72]	-0.72 [-0.96, -0.48]*
Intermediate	0.50 [0.35, 0.73]*	1.19 [0.82, 1.74]	0.24 [0.17, 0.33]*	2.25 [0.47, 10.87]	-1.63 [-1.88, -1.38]*
Small employers	0.19 [0.10, 0.36]*	1.40 [0.94, 2.11]	0.24 [0.16, 0.35]*	1.10 [0.15, 7.86]	-2.05 [-2.34, -1.77]*
Lower supervisory	0.35 [0.22, 0.56]*	1.36 [0.91, 2.02]	0.12 [0.08, 0.20]*	2.78 [0.56, 13.84]	-2.32 [-2.60, -2.04]*
Semi-routine	0.23 [0.14, 0.39]*	1.60 [1.11, 2.32]*	0.10 [0.06, 0.15]*	3.38 [0.73, 15.74]	-2.53 [-2.79, -2.27]*
Routine	0.22 [0.13, 0.39]*	1.12 [0.74, 1.69]	0.04 [0.02, 0.08]*	3.81 [0.80, 18.05]	-2.74 [-3.02, -2.46]*
	Highest qualification				
	NVQ1 level vs None	NVQ2 level vs None	NVQ3 level vs None	NVQ4 level vs None	NVQ5 level vs None
	OR [95% CI]	OR [95% CI]	OR [95% CI]	OR [95% CI]	OR [95% CI]
Higher managerial	1.00 [Reference]	1.00 [Reference]	1.00 [Reference]	1.00 [Reference]	1.00 [Reference]
Lower managerial	0.91 [0.52, 1.58]	1.09 [0.73, 1.64]	0.79 [0.52, 1.20]	0.67 [0.46, 0.98]*	0.62 [0.41, 0.94]*
Intermediate	1.57 [0.90, 2.74]	1.61 [1.05, 2.46]*	1.00 [0.65, 1.55]	0.74 [0.50, 1.09]	0.52 [0.33, 0.82]*
Small employers	1.20 [0.68, 2.13]	1.06 [0.68, 1.63]	0.75 [0.48, 1.17]	0.37 [0.24, 0.55]*	0.23 [0.14, 0.37]*
Lower supervisory	1.06 [0.61, 1.84]	0.93 [0.61, 1.41]	0.64 [0.42, 0.98]*	0.32 [0.22, 0.47]*	0.14 [0.08, 0.23]*
Semi-routine	1.30 [0.78, 2.17]	0.80 [0.54, 1.19]	0.42 [0.28, 0.63]*	0.23 [0.16, 0.32]*	0.10 [0.06, 0.17]*
Routine	1.08 [0.63, 1.83]	0.73 [0.49, 1.10]	0.42 [0.27, 0.64]*	0.20 [0.13, 0.29]*	0.09 [0.05, 0.15]*
	Status of institution		Field of study		
	Normal status vs No degree	Higher status vs No degree	STEM vs No degree	LEM vs No degree	OSSAH vs No degree
	OR [95% CI]	OR [95% CI]	OR [95% CI]	OR [95% CI]	OR [95% CI]
Higher managerial	1.00 [Reference]	1.00 [Reference]	1.00 [Reference]	1.00 [Reference]	1.00 [Reference]
Lower managerial	0.70 [0.57, 0.85]*	0.53 [0.41, 0.68]*	0.58 [0.46, 0.73]*	0.70 [0.51, 0.97]*	0.68 [0.52, 0.87]*
Intermediate	0.47 [0.38, 0.58]*	0.29 [0.21, 0.39]*	0.40 [0.32, 0.51]*	0.42 [0.30, 0.61]*	0.38 [0.29, 0.51]*
Small employers	0.34 [0.26, 0.44]*	0.15 [0.10, 0.23]*	0.26 [0.19, 0.36]*	0.32 [0.20, 0.50]*	0.25 [0.17, 0.36]*
Lower supervisory	0.28 [0.22, 0.36]*	0.15 [0.10, 0.22]*	0.22 [0.16, 0.30]*	0.30 [0.20, 0.47]*	0.21 [0.14, 0.31]*
Semi-routine	0.24 [0.19, 0.31]*	0.11 [0.08, 0.17]*	0.20 [0.15, 0.27]*	0.23 [0.15, 0.35]*	0.17 [0.12, 0.24]*
Routine	0.22 [0.17, 0.30]*	0.08 [0.05, 0.14]*	0.20 [0.14, 0.27]*	0.16 [0.09, 0.27]*	0.15 [0.09, 0.22]*

*NS-SEC* National statistics socioeconomic classification

Multinomial logistic regression was fitted for categorical measures of education (school type at age 16, highest qualification, institution status and field of study) Odds ratios (OR) were reported

Linear regression was fitted for numerical measures of education (age left full-time education). Unstandardized regression coefficients (Coef.) were reported

All estimates were adjusted for confounders (sex, ethnicity, country of residence and residence area)

^*^p < 0.05

**Table 3:** Models for the association between various measures of education and poor subjective oral health (*n* = 6703)

	**Poor subjective oral health**		**Crude association**	**Adjusted association**
	**n**	**%**	**OR [95% CI]**	**OR [95% CI]**
*School type at age 16 years*				
Comprehensive	1332	24.3	1.00 [Reference]	1.00 [Reference]
Grammar	44	16.8	0.63 [0.45, 0.88]*	0.71 [0.51, 0.99]*
Secondary Modern	118	24.4	1.01 [0.81, 1.25]	0.99 [0.79, 1.23]
Private	73	17.1	0.64 [0.50, 0.83]*	0.76 [0.58, 0.99]*
Special Needs	15	35.7	1.73 [0.92, 3.27]	1.48 [0.78, 2.81]
*Highest qualification*				
None	215	36.3	1.00 [Reference]	1.00 [Reference]
NVQ1 level	142	29.5	0.73 [0.57, 0.95]*	0.74 [0.57, 0.96]*
NVQ2 level	441	26.9	0.65 [0.53, 0.79]*	0.67 [0.55, 0.83]*
NVQ3 level	258	25.5	0.60 [0.48, 0.75]*	0.63 [0.50, 0.78]*
NVQ4 level	448	18.6	0.40 [0.33, 0.49]*	0.45 [0.37, 0.55]*
NVQ5 level	78	13.9	0.28 [0.21, 0.38]*	0.33 [0.25, 0.45]*
*Institution status*				
No degree	1319	26.1	1.00 [Reference]	1.00 [Reference]
Normal status	195	16.6	0.56 [0.48, 0.66]*	0.62 [0.52, 0.73]*
Higher status	68	14.2	0.47 [0.36, 0.61]*	0.54 [0.41, 0.70]*
*Field of study*				
No degree	1319	26.1	1.00 [Reference]	1.00 [Reference]
STEM	126	16.2	0.55 [0.45, 0.67]*	0.58 [0.47, 0.71]*
LEM	46	13.9	0.46 [0.33, 0.63]*	0.50 [0.36, 0.69]*
OSSAH	91	16.6	0.56 [0.44, 0.71]*	0.69 [0.54, 0.87]*
*Age left full time education*	-	-	0.91 [0.90, 0.93]*	0.93 [0.91, 0.95]*

Binary logistic regression models were fitted, and odds ratios (OR) reported

Adjusted models were controlled for sex, ethnicity, country of residence, residence area and NS-SEC group

^*^p < 0.05

Having established that the measures of education were both associated with both parental SES and subjective oral health, their mediating effect between exposure and outcome was evaluated individually and jointly in Table 4. The effect of parental SES on subjective oral health was partially mediated by each measure of education, with a proportion mediated of 53.2% for the institution status, 46.5% for the field of study, 42.8% for the school type, 38.9% for the highest qualification earned and 38.4% for the age when full-time education was discontinued. The proportion of the effect of parental SES on subjective oral health jointly mediated by all measures of education was 81.1%, which was substantially larger than the individual effect of any measure of education.

**Table 4:** Total, natural direct and indirect effects of parental SES on poor subjective oral health (*n* = 6703)

Mediators	TE	NIE	NDE	Proportion mediated
	**Parental SES → oral health**	**Parental SES → mediator → oral health**	**Parental SES → other pathways → oral health**	
	**OR [95% CI]**	**OR [95% CI]**	**OR [95% CI]**	**% [95% CI]**
School type at age 16 years	1.99 [1.58, 2.81]*	1.27 [1.15, 1.40]*	1.92 [1.51, 2.43]*	42.8 [22.6, 62.6]*
Highest qualification	1.99 [1.60, 2.51]*	1.24 [1.10, 1.41]*	1.88 [1.47, 2.43]*	38.9 [19.3, 68.6]*
Institution status	1.99 [1.62, 2.60]*	1.36 [1.16, 1.58]*	1.72 [1.34, 2.22]*	53.2 [27.3, 83.1]*
Field of study	1.99 [1.52, 2.45]*	1.30 [1.12, 1.52]*	1.72 [1.33, 2.18]*	46.5 [22.2, 84.2]*
Age left full-time education	1.99 [1.59, 2.48]*	1.24 [1.04, 1.55]*	1.70 [1.28, 2.21]*	38.4 [6.9, 80.7]*
All mediators jointly	1.99 [1.61, 2.51]*	1.68 [1.32, 1.98]*	1.75 [1.39, 2.22]*	81.1 [48.1, 116.3]*

*NDE* Natural direct effect; NIE: natural indirect effect, *TE* total effect, *CI* confidence intervals

Odds ratios (OR) for total, direct and indirect effects indicate the odds of poor subjective oral health for individuals in the lowest parental SES group relative to those for individuals in the highest parental SES group

^*^p < 0.05

Sensitivity analysis regarding the role of unmeasured confounding showed that the observed NDE could be explained away by an unmeasured confounder that was associated with both the exposure and outcome by risk ratios of 2.08-fold each, above and beyond the measured confounders, but weaker confounding could not do so. In addition, an unmeasured confounder associated with the mediator and outcome with approximate risk ratios of 1.92-fold each would suffice to completely explained away the observed NIE, above and beyond the measured confounders, but weaker confounding would not.

## Discussion

The findings of this study supported the mediating role of education in the association between parental SES and subjective oral health in middle adulthood. However, they also showed that inequalities in adult oral health between parental SES groups remained, to some extent, after accounting for education measures and demographic factors, suggesting that alternative pathways may be in place.

In line with the social pathway model [7], we found that individuals raised in higher socioeconomic backgrounds were more likely to have educational opportunities that were associated with lower odds of reporting poor oral health later in life. Education mediated about 81% of the association between parental SES and adult oral health. These findings demonstrate that individuals, including those raised in low socioeconomic conditions, can gain through education the skills underpinning healthy lifestyles and access to preventive care [11, 13]. However, although education is a well-established determinant of oral health, our findings also highlight that these educational opportunities, from high school to college/university, are determined by early socioeconomic circumstances. The cohort members of the BCS70 have experienced free compulsory (comprehensive schools are for children of all abilities) and higher education (universities were public and charged no tuition fees at the time). Aside from private schools, access to selective schools (Grammars) and university entrance requirements were largely based on examinations [27]. All five measures of education were associated with oral health in models adjusted for demographic factors and parental SES. These findings imply that using the highest qualification earned as a single indicator may oversimplify the relationship between education and health. In the present study, the highest qualification earned explained 38.9% of the exposure-outcome association only. Indeed, recent evidence suggests that the educational trajectories that individuals follow over their lifespan seem important to health [21, 36]. Understanding these relations may shed lights on the relevance of different facets of education to oral health.

Although education mediated a substantial proportion of the association between parental SES and adult oral health, it did not fully explain it. This finding indicates that parental SES has long-term effects on oral health and that there may be other pathways from childhood socioeconomic circumstances to adult health besides education. Individuals whose parents were in the lowest parental SES group had around 99% greater odds of reporting poor oral health. The long arm of childhood SES can be explained by the materialistic and behavioural pathways to health. Parents from high SES are more likely to access preventive and curative dental services. Dental caries, the most common childhood oral disease, follows trajectories from childhood to adulthood [37]. Therefore, by accessing preventive and curative dental services early in life, parents in higher SES groups may be placing their children in lower caries trajectories than those in lower SES groups. It is also during childhood that health behaviours, such as a diet low in added sugars, frequent toothbrushing and regular dental recalls, are set within the family environment with parents acting as role models as well as supervising the initiation and maintenance of such behaviours [38].

Our findings have some implications. Education is the dominant factor in explaining the link between social origins and destinations, and thus, it is seen as the main vehicle for intergenerational social mobility [8, 9]. Strengthening free public education, for instance through educational expansion and improving education quality and retention, are plausible policies to achieve the goal of increasing social mobility and, subsequently, reducing social inequalities in health. Richer analysis on educational trajectories could provide valuable information with relevance for health outcomes later in life. These detailed analyses can suggest which facets of education to target for intervention.

This study has some limitations. First, our outcome measure was self-reported. However, using a single-global item is a cost-effective method in large population studies as it provides a valid reflection of oral health status [39]. Second, around a fifth of participants in BCS70 wave 10 were excluded from the analysis due to missing data (mostly for parental SES). As individuals in the study sample had higher parental SES and education and reported better oral health, this could have introduced selection bias and underestimated the associations of parental SES and education with adult oral health. Third, although we had a clear temporal ordering between exposure, mediators and outcome, we considered confounders at baseline only. This is the simplest scenario in casual mediation analysis and a simplification of a more complex reality, which likely includes time-varying confounders of the mediator-outcome relationship, especially variations in parental SES from childhood to adulthood. Finally, valid estimation in causal mediation analysis requires that unmeasured confounding assumptions be met [29, 31]. Parental health status could be a potential confounder of the exposure-outcome association whereas cohort members’ intellectual ability and health status in early life could confound the mediator-outcome association. However, our sensitivity analysis suggested that any unmeasured confounder will need to be strongly associated with the exposure (or mediator) and outcome for the observed NDE and NIE to be fully explained away. Further research is needed to confirm the present findings and generalize them to other cohorts and countries with different educational systems.

## Conclusion

This analysis of the British Cohort Study 1970 revealed that multiple measures of education explained a substantial proportion of the association between parental SES and subjective oral health in middle adulthood. These findings suggest that parental SES has both a direct effect on adult oral health as well as an indirect effect via education.

## Data Availability

The data for this study are available from the UK Data Archive upon registration.

## Abbreviations

BCS70: British Cohort Study 1970
LEM: Law, Economics, Management
NS-SEC: National Statistics Socio-Economic Classification
NVQ: National Vocational Qualification
OSSAH: Other Social Sciences, Arts and Humanities
SES: Socioeconomic status
STEM: Science, Technology, Engineering, Maths
